# A particle filter for ammonia coverage ratio and input simultaneous estimations in Diesel-engine SCR system

**DOI:** 10.1371/journal.pone.0192217

**Published:** 2018-02-06

**Authors:** Kangfeng Sun, Fenzhu Ji, Xiaoyu Yan, Kai Jiang, Shichun Yang

**Affiliations:** School of Transportation Science and Engineering, Beihang University, Beijing, China; Chongqing University, CHINA

## Abstract

As NOx emissions legislation for Diesel-engines is becoming more stringent than ever before, an aftertreatment system has been widely used in many countries. Specifically, to reduce the NOx emissions, a selective catalytic reduction(SCR) system has become one of the most promising techniques for Diesel-engine vehicle applications. In the SCR system, input ammonia concentration and ammonia coverage ratio are regarded as essential states in the control-oriental model. Currently, an ammonia sensor placed before the SCR Can is a good strategy for the input ammonia concentration value. However, physical sensor would increase the SCR system cost and the ammonia coverage ratio information cannot be directly measured by physical sensor. Aiming to tackle this problem, an observer based on particle filter(PF) is investigated to estimate the input ammonia concentration and ammonia coverage ratio. Simulation results through the experimentally-validated full vehicle simulator cX-Emission show that the performance of observer based on PF is outstanding, and the estimation error is very small.

## Introduction

In recent years, a phenomenon is that Diesel-engines have got more attentions due to their excellent fuel efficiency and lower greenhouse emissions compared with gasoline counterparts[1]. And Diesel-engines are quite important for the conventional and electrified vehicles in the future[2]. However, the Diesel-engine flame temperatures are high, which lead to the oxidation of nitrogen into NOx[3, 4]. The emissions of NOx would result to ozone depletion, acid rain and photochemical smog formation, which do harm to the environment and human health[4, 5]. To avoid NOx emissions, the legislation for Diesel-engines emissions is becoming increasingly stringent than ever before. In order to meet with the current and proposed emission regulations, many efforts have been paid to deal with the high-NOx emissions problems[6]. It is known that only employing combustion control strategies such as variable valve actuation system(VVA), exhaust gas recirculation(EGR) etc. cannot meet the stringent emission regulations[5, 7, 8]. Therefore, some types of aftertreatment systems including the diesel oxidation catalyst(DOC), the diesel particle filter(DPF), and the selective catalytic reduction(SCR) systems are widely used in the Diesel-engines. Specifically, the main function of the DOC system is to oxidize the un-burned hydrocarbon and the carbon monoxide. The DPF device is used to capture the PM. The SCR component has the ability to convert the NOx emissions into nitrogen (N_2_) and water (H_2_O) which are friendly to the environment and human health. Among all types of aftertreatment systems, pointed in Ref. [9], the urea-based SCR system has been proved one of the most promising techniques to reduce NOx emissions and fulfill NOx emissions standards for Diesel-engine vehicle applications in the future.

In the urea-based SCR system, the primary principle is to spray urea solution into the exhaust pipe before the SCR tank. Under the high temperature exhaust condition, the injected urea solution can be converted into gaseous ammonia which is then absorbed on the catalyst surface and reacts with NOx[10]. Generally speaking, higher urea injection would lead to higher NOx conversion and less NOx emissions in the tailpipe[3]. However, if the urea is over dosed than the NOx emissions, the gaseous ammonia would slip into the tailpipe which is also harmful to the environment and human health. Thus, the urea dosing closed-loop control, aiming to achieve a high NOx conversion and low ammonia slip, of the SCR system, is proposed[11]. Many studies on the urea dosage control can be seen in[12, 13].

Among the SCR closed-loop control strategies, the input ammonia concentration and the ammonia coverage ratio are considered as the critical states in the construction of the feedback control strategy[2, 14]. To obtain the input ammonia concentration information, a physical sensor placed at the inlet of the SCR system is utilized to measure the concentration value. However, on one hand, the physical commercial sensors would increase the SCR cost and diagnosis challenge[15, 16]. On the other hand, there is no commercial sensor to measure the ammonia coverage ratio directly[3]. To deal with this problem, a promising strategy is to design an observer to estimate the input ammonia concentration and ammonia coverage ratio information. In order to develop the observer, there are considerable methods. The well-known strategies include the Kalman filter(KF)[17] and the Luenberger observer[18] which have been successfully applied to many fields of science and engineering. However, both methods are suitable for linear systems. It is important to note that the chemical processes in the SCR tank are complex and the approximated SCR model exhibits strong nonlinear dynamics and possible non-Gaussian noise[9, 19]. Therefore, how to develop an observer that provides an exact optimal solution to the SCR system remains an open problem. Though the modified KF, also known as extended Kalman filter(EKF), is the most traditional estimator for nonlinear systems, it has been demonstrated to perform poorly in the non-Gaussian posterior estimates obtained when the system is nonlinear[20]. The EKF requires that the nonlinear state transition equations and measurement equations are approximated to linear equations by using the first-order Taylor series expansion, so that the KF can be applied to estimate the states. Moreover, it requires the nonlinearity of the model is small and the deviation of the approximation is not big[20], which is however not exhibited by the SCR system that involves complicate chemical reaction processes and time-varying reaction conditions. Therefore, though the EKF works well in many cases, an alternate observer needs to be developed to provide a better estimation in the SCR system. To address the uncertainties especially in strong nonlinear and non-Gaussian systems, particle filter(PF) algorithm has been given relatively more efforts. Unlike the EKF, PF relies on fitting an approximate solution to the nonlinear equation rather than solving the linear equations obtained by approximating the nonlinear system[20]. It makes use of the Bayesian filter and Monte Carlo method to estimate the states. The state distribution is represented by a set of random samples with associated weights. High probability density is represented by a huge number of particles, low probability by a low number or no particles[20]. It refers from many researchers that for strong nonlinear and non-Gaussian systems, if the model is not accurate enough, then the PF algorithm is considered very useful to do the state estimation[21, 22]. A well-studied applications of PF is the lithium-ion battery system, where PFs were used for the state estimation such as state-of-health, state of charge, remaining useful life, etc.[23–25]. Therefore, in order to deal with the strong nonlinear dynamics and possible strong non-Gaussian noise in the SCR system, we aim to design an observer based on the PF to estimate both ammonia inputs and coverage ratio at the same time.

In this paper, we propose a technique by using the PF to simultaneously estimate the ammonia input and ammonia coverage ratio. As a contrast, an observer based on the EKF is also designed. The contributions and novelty of the work can be summarized as: (1) Considering the strong nonlinearity, it aims to propose the PF algorithm for the SCR system. (2) Based on the PF, the observer is designed to estimate the ammonia coverage ratio which is a crucial state and cannot be directly measured by physical sensors, and the input ammonia concentration is estimated such that the urea-to-ammonia rate is available without mounting an extra ammonia sensor at the entrance of the SCR tank. (3) The proposed PF observer is verified by using the data from an experimentally-validated full vehicle simulator cX-Emission. The comparisons show the advantage of the proposed PF observer over the traditional EKF observer.

The rest of this paper is organized as follows. A concise introduction of the SCR reaction model is shown in section 2. Then an observer model for state estimation is designed in section 3. In section 4, the simulation results are presented to verify the performance of the proposed technique. At last, concluding remarks are summarized in section 5.

## Selective catalytic reduction(SCR)

### 2.1. SCR operation principle

**Fig 1:**
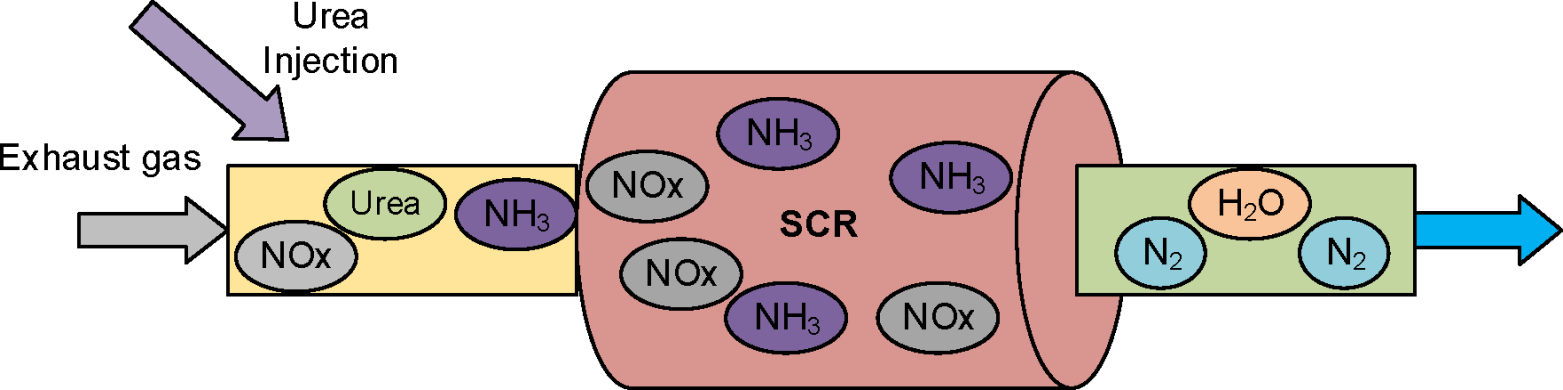
Schematic diagram of SCR system.

The main function of a SCR system is to catalytically convert the engine-out NOx into nitrogen and water, and consequently, reduce the NOx emissions in the exhaust tailpipe of a Diesel-engine. Therefore, in the urea-based SCR system, the 32.5% aqueous urea solution is injected into the upstream exhaust pipe. The schematic diagram of a SCR system is shown in Fig 1. Generally speaking, the NOx reduction mainly consists of three parts. (1) urea solution sprayed into the exhaust pipe is converted into ammonia through a certain number of processes including the evaporation of urea solution, thermal decomposition of pure urea, and the hydrolyzation of isocyanic acid. Then, the gaseous ammonia flows into upstream of the SCR device. (2) most of the ammonia is absorbed on the surface of the catalyst. Meanwhile, the absorbed ammonia is partly desorbed from the surface. (3) the absorbed ammonia reacts with NOx and converts it into nitrogen and water[26, 27].

The chief chemical reactions and corresponding reaction rate equations are summarized as follows:

(1) Urea evaporation: the urea solution evaporates into solid phase of urea under the ideal situation. The reaction is represented as follows:
  $${\mathrm{NH}}_{2}‑\mathrm{CO}‑{\mathrm{NH}}_{2}(\mathrm{liquid})\to {\mathrm{NH}}_{2}‑\mathrm{CO}‑{\mathrm{NH}}_{2}^{*}+6.9H_{2}O$$ (1)
  where ${\mathrm{NH}}_{2}‑\mathrm{CO}‑{\mathrm{NH}}_{2}^{*}$ expresses the pure urea.
(2) Urea decomposition: solid urea is decomposed into ammonia and equimolar amount of isocyanic acid when the exhaust gas temperature is sufficiently high. The reaction is shown as follows:
  $${\mathrm{NH}}_{2}‑\mathrm{CO}‑{\mathrm{NH}}_{2}^{*}\to \mathrm{HNCO}+{\mathrm{NH}}_{3}$$ (2)
(3) Isocyanic acid hydrolyzation: in this part, the isocyanic acid is hydrolyzed to ammonia and carbon dioxide. The reaction is described as follows:
  $$\mathrm{HNCO}+H_{2}O\to {\mathrm{NH}}_{3}+{\mathrm{CO}}_{2}$$ (3)
(4) Adsorption/desorption: the ammonia in the catalytic tank is adsorbed on the SCR substrate, and the adsorbed ammonia can also be desorbed from the substrate. The reversible reaction is represented as follows:
  $${\mathrm{NH}}_{3}+\theta_{\mathrm{free}}⇌{\mathrm{NH}}_{3}^{*}$$ (4)
  Where θ_free_ is the free SCR catalyst site, and the ${\mathrm{NH}}_{3}^{*}$ refers to the NH_3_ adsorbed on the SCR substrate which is ready to react with NOx; the forward direction describes the adsorption process while the reverse direction denotes the desorption process.

The rate of the revisable reaction can be shown as follows[28]:

$$R_{\mathrm{ad}}=K_{\mathrm{ad}}\exp(‑\frac{E_{\mathrm{ad}}}{\mathrm{RT}})C_{{\mathrm{NH}}_{3}}(1‑\theta_{{\mathrm{NH}}_{3}})$$ (5)

$$R_{\mathrm{de}}=K_{\mathrm{de}}\exp(‑\frac{E_{\mathrm{de}}}{\mathrm{RT}})C_{{\mathrm{NH}}_{3}}\theta_{{\mathrm{NH}}_{3}}$$ (6)

where Rx represents the rate of the chemical reaction; T is temperature; E, K, and R are constants; Cx expresses concentration of x, and $\theta_{{\mathrm{NH}}_{3}}$ is the ammonia coverage ratio, which is defined as:

$$\theta_{{\mathrm{NH}}_{3}}=\frac{M_{{\mathrm{NH}}_{3}}^{*}}{\Theta }$$ (7)

where $M_{{\mathrm{NH}}_{3}}^{*}$ indicates the number of moles of the ammonia adsorbed on the SCR substrate. Θ expresses the ammonia storage capacity which depends on the exhaust temperature.

(5) NOx reduction: the chief reduction reactions can be described as follows:
  $$4{\mathrm{NH}}_{3}^{*}+4\mathrm{NO}+O_{2}\to 4N_{2}+6H_{2}O$$ (8)
  $$2{\mathrm{NH}}_{3}^{*}+\mathrm{NO}+NO_{2}\to 2N_{2}+3H_{2}O$$ (9)
  $$4{\mathrm{NH}}_{3}^{*}+3NO_{2}\to 3.5N_{2}+6H_{2}O$$ (10)

Among the Diesel-engine exhaust NOx, NO is more than 90%, and the reaction rate of Eq (8) is quite fast. Therefore, in the SCR reaction tank, the dominant reduction reaction is Eq (8). The relevant reaction rate is shown as follows:

$$R_{\mathrm{re}}=K_{\mathrm{re}}\exp(‑\frac{E_{\mathrm{re}}}{\mathrm{RT}})C_{\mathrm{NO}}\theta_{{\mathrm{NH}}_{3}}$$ (11)

(6) Ammonia oxidation: if the temperature is more than 450°C, the adsorbed NH_3_ would be oxidized to NO. The reaction equation can be proposed as follows:
  $${\mathrm{NH}}_{3}^{*}+1.25O_{2}\to \mathrm{NO}+1.25H_{2}O$$ (12)

The reaction rate is presented as below:

$$R_{\mathrm{ox}}=K_{\mathrm{ox}}\exp(‑\frac{E_{\mathrm{ox}}}{\mathrm{RT}})\theta_{{\mathrm{NH}}_{3}}$$ (13)

### 2.2. SCR model

In this work, we suppose that the SCR reaction device is a Continuously Stirred Tank Reactor (CSTR). Based on the above chemical reactions, molar balance and the conservation of mass, the dynamic equation of SCR system can be established as follows.

$${\dot{C}}_{\mathrm{NO}}=\Theta (R_{\mathrm{ox}}‑R_{\mathrm{re}})$$ (14)

$${\dot{\theta }}_{{\mathrm{NH}}_{3}}=R_{\mathrm{ad}}‑R_{\mathrm{de}}‑R_{\mathrm{re}}‑R_{\mathrm{ox}}$$ (15)

$${\dot{C}}_{{\mathrm{NH}}_{3}}=\Theta (R_{\mathrm{de}}‑R_{\mathrm{ad}})$$ (16)

In the literature, there are many SCR models for ground vehicle applications such as the four-state model in[29], and five-state model in[10]. Generally speaking, four states or more states model may describe the actual SCR system better. However, with four or more states, the model designing and parameter identification would be more complicated. What’s more, to design controllers, detect fault and monitor system, more sensors and observers are also needed[30]. Therefore, considering the model accuracy and the project cost, a three-state nonlinear model is developed as below, which has been validated by Diesel-engine aftertreatment experiments[31, 32]:

$$\left[\begin{array}{c} {\dot{C}}_{\mathrm{NO}}\\ {\dot{\theta }}_{{\mathrm{NH}}_{3}}\\ {\dot{C}}_{{\mathrm{NH}}_{3}} \end{array}\right]=\left[\begin{array}{c} ‑C_{\mathrm{NO}}(\Theta r_{\mathrm{re}}\theta_{{\mathrm{NH}}_{3}}+\frac{F}{V})+r_{\mathrm{ox}}\Theta \theta_{{\mathrm{NH}}_{3}}\\ ‑\theta_{{\mathrm{NH}}_{3}}(r_{\mathrm{ad}}C_{{\mathrm{NH}}_{3}}+r_{\mathrm{de}}+r_{\mathrm{re}}C_{\mathrm{NO}}+r_{\mathrm{ox}})+r_{\mathrm{ad}}C_{{\mathrm{NH}}_{3}}\\ ‑C_{{\mathrm{NH}}_{3}}\left[\Theta r_{\mathrm{ad}}(1‑\theta_{{\mathrm{NH}}_{3}})+\frac{F}{V}\right]+r_{\mathrm{de}}\Theta \theta_{{\mathrm{NH}}_{3}} \end{array}\right]+\left[\begin{array}{c} 0\\ 0\\ \frac{F}{V} \end{array}\right]C_{{\mathrm{NH}}_{3},\mathrm{in}}+\left[\begin{array}{c} \frac{F}{V}\\ 0\\ 0 \end{array}\right]C_{\mathrm{NO},\mathrm{in}}$$ (17)

where $r_{x}=K_{x}\exp(‑\frac{E_{x}}{\mathrm{RT}})$, x denotes ad, de, ox, re; C_NO_ and $C_{{\mathrm{NH}}_{3}}$ represent the NOx and ammonia concentration in the tailpipe; $C_{{\mathrm{NH}}_{3},\mathrm{in}}$ and C_NO,in_ indicates the inlet ammonia and NOx concentration respectively. F is the exhaust gas flow rate; V is the volume of the SCR Can.

## Observer design for ammonia input and coverage ratio estimations

**Fig 2:**
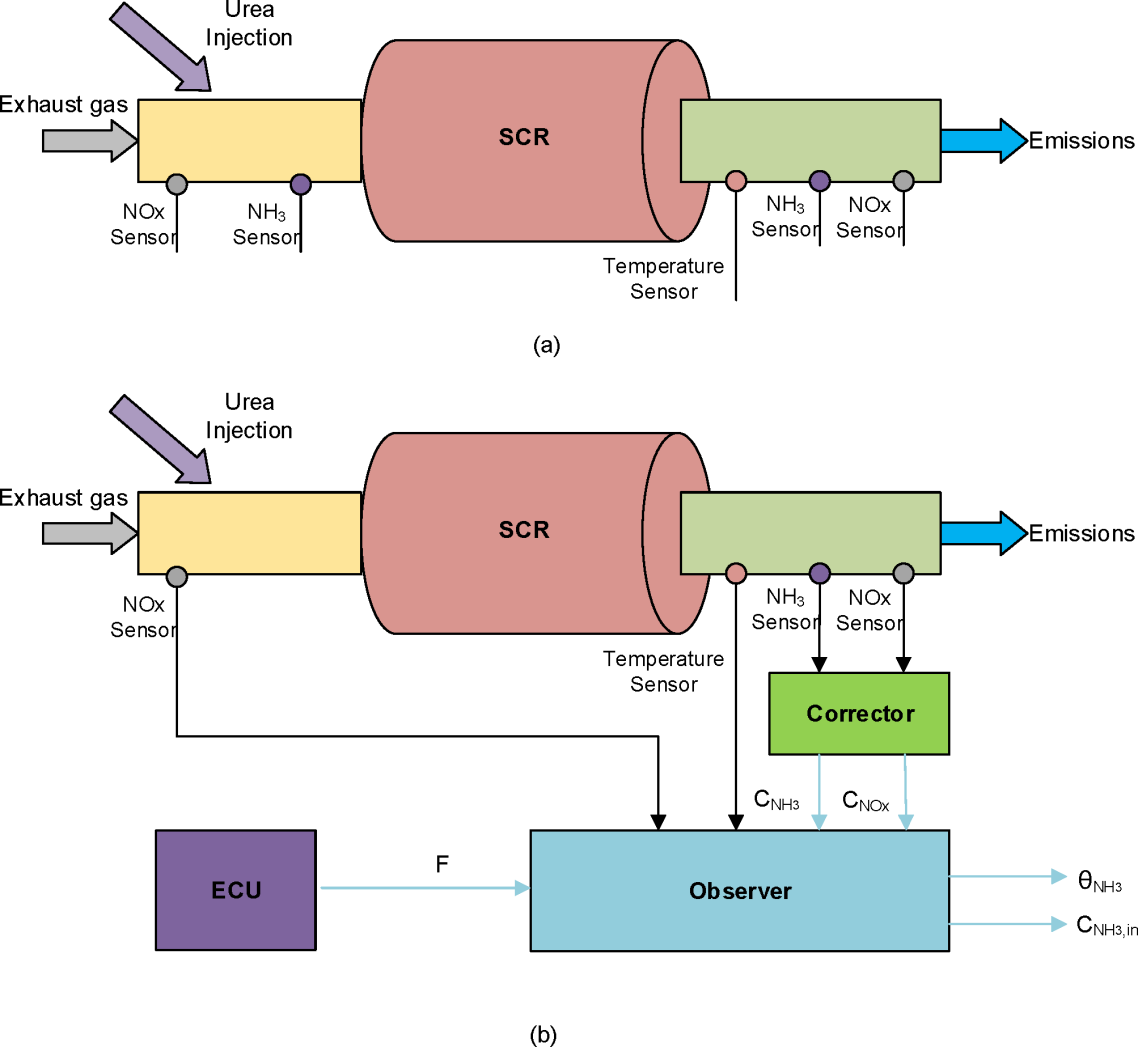
Schematic diagram of SCR system with corresponding sensors (a) and with observer architecture diagram (b).

In appropriate SCR controllers, input concentrations, state information, output concentrations and temperature are necessary. In general, two ammonia sensors, two NOx sensors and one temperature sensor are mounted in the SCR device to measure the essential parameters. The schematic diagram of the SCR system and location of sensors are presented in Fig 2(A). Actually, the physical sensors are expensive and too many sensors would increase the SCR cost and diagnosis challenge. Moreover, there is no commercial sensor to measure the ammonia coverage ratio directly which is a crucial state in the construction of the feedback control strategy. Thus, it is necessary to design an observer to estimate the ammonia coverage ratio and input ammonia concentration. The architecture diagram of the observer designed in this work is shown in Fig 2(B) which estimates the ammonia coverage ratio and reduces an ammonia sensor at the entrance of the SCR tank.

In order to develop the observer, there are considerable methods. Considering the system nonlinearities and measurement noises, the EKF algorithm is the most classic and widely-used method. However, the method requires that the linearization of the nonlinear model to which the KF can be applied keeps small deviation, the nonlinearity of the model is not big and the noise is Gaussian or independent and identically distributed which are hard to satisfy in the SCR system[19, 20]. Therefore, the observer based on EKF algorithm may not perform well in the state estimation of the SCR system. Considering the strong uncertainties in the SCR system, PF algorithm is a novel filtering method which fits on an approximate solution to the nonlinear model rather than solving the linear model obtained by approximating the nonlinear system[20, 33]. More importantly, PF algorithm should have notably better estimation accuracy than EKF approach. It makes use of the recursive Bayesian filtering of Monte Carlo simulations towards state estimation and makes no restriction to the state space models and the distribution of the noise.

In our study, the traditional EKF and PF have been used to estimate the ammonia input concentration and coverage ratio. The algorithms for the two observers are described in the following sections. Then the state space model for observer is presented.

### 3.1. The EKF algorithsm

The EKF algorithm is the most widely used state estimation technique in nonlinear system which uses a linearized model and KF algorithm at each sample time. The nonlinear system can be described as below:

$$x_{k}=f[x_{k‑1},u_{k}]+w_{k}$$ (18)

$$z_{k}=h[x_{k}]+v_{k}$$ (19)

where *x*_*k*_ and *u*_*k*_ is the state vector and input vector respectively, f(x) expresses the relevant prediction function, *w*_*k*_ denotes the process noise whose covariance is *Q*_*k*_. In (19), h(x) is the measurement function, the corresponding noise and noise covariance are *v*_*k*_ and *R*_*k*_ respectively.

Following a linearization procedure of Eq (18) and Eq (19), linear state space form to which KF can be applied is obtained as follows:

$$x_{k+1}=A_{k}x_{k}+B_{k}U_{k}+w_{k}$$ (20)

$$z_{k}=H_{k}x_{k}+v_{k}$$ (21)

Then the EKF recursion can be summarized below.

(1) Prediction step: the priori estimations of the state vector *x*_*k*_ and the system error covariance *P*_*k*_ are obtained as follows.
  $${\hat{x}}_{k}^{-}=f({\hat{x}}_{k-1},u_{k-1})$$ (22)
  $$P_{k}^{-}=A_{k-1}P_{k-1}A_{k-1}^{T}+Q$$ (23)
(2) Calculate the Kalman gain: the Kalman gain defines the extent to which the observer trust the state estimate or measurement[20].
  $$K_{k}=P_{k}^{-}H_{k-1}^{T}{\left(H_{k}P_{k}^{-}H_{k}^{T}+R\right)}^{-1}$$ (24)
(3) Measurement update step: calculate the posteriori estimations of the state vector and error covariance using the measurement information.
  $${\hat{x}}_{k}={\hat{x}}_{k}^{-}{+K}_{k}(z_{k}-h({\hat{x}}_{k}^{-}))$$ (25)
  $${P_{k}=P}_{k}^{-}-K_{k}H_{k}P_{k}^{-}$$ (26)

### 3.2. The PF algorithm

Unlike the EKF which requires the linearization of the nonlinear system, the PF algorithm does not require that the system is linear or Gaussian distributed by using the Bayesian filter and Monte Carlo approach. This method begins with a set of random particles which are then propagated through the state transition equation to obtain priori particles. Then, the available measurement information is fused with these priori particles to give posteriori distribution. An excellent tutorials of PF can be seen in [34]. For a nonlinear system described in Eq (18) and Eq (19), the PF algorithm can be performed in 3 steps.

(1) Initialization: a set of random particles *x*_0,*i*_(i = 1,2,⋯,N) are generated based on the initial pdf of p(*x*_0_).
(2) Importance sampling: the randomly generated particles are propagated through the state transition equation Eq (18) to obtain the priori distribution particles with the importance weight *q*_*i*_ which can be calculated as follows.
  $$x_{k,i}^{-}=f(x_{k-1,i},u_{k-1})+w_{k-1}(i=1,2,\cdots ,N)$$ (27)
  $$q_{i}=p\langle \left(z_{k}=z^{*}\right)|\left(x_{k}=x_{k,i}^{-}\right)\rangle =p[v_{k}=z^{*}-h(x_{k,i}^{-})]\approx \frac{1}{({2\pi )}^{m/2}{\left|R\right|}^{1/2}}exp(\frac{-{\left[z^{*}-h(x_{k,i}^{-})\right]}^{T}R^{-1}[z^{*}-h(x_{k,i}^{-})]}{2})$$ (28)
  $$q_{i}=\frac{q_{i}}{\sum_{j=1}^{N}q_{j}}$$ (29)
  where z* indicates the measurement value.
(3) Resampling: resampling approach are made to reduce the degeneracy phenomena during the iteration. After resampling, the particles with large weight are replicated and particles with small weight are discarded[33]. There are several methods to make resampling, such as systematic resampling, multinational resampling, residual sampling, etc.[35]. In this paper, the systematic resampling

### 3.3. Ammonia input and coverage ratio estimations

Since the system model is built and the necessary parameters can be measured by physical sensors, the input ammonia concentration and ammonia coverage ratio can be estimated by EKF and PF described above. As the ammonia concentration varies very slowly over time, then, the derivative of input ammonia concentration can be assumed as zero[30]:

$${\dot{C}}_{{\mathrm{NH}}_{3},\mathrm{in}}=0$$ (30)

The state space model in discrete form for input ammonia concentration and ammonia coverage ratio predictions are built as follows:

$$\hat{x}(k|k‑1)=\left[\begin{array}{c} {\hat{\theta }}_{{\mathrm{NH}}_{3}}(k|k‑1)\\ {\hat{C}}_{{\mathrm{NH}}_{3},\mathrm{in}}(k|k‑1) \end{array}\right]=f\left[\hat{x}(k‑1|k‑1)\right]=\left[\begin{array}{c} {\hat{\theta }}_{{\mathrm{NH}}_{3}}(k‑1|k‑1)+∆T{\dot{\hat{\theta }}}_{{\mathrm{NH}}_{3}}(k|k‑1)\\ {\hat{C}}_{{\mathrm{NH}}_{3},\mathrm{in}}(k‑1|k‑1) \end{array}\right]$$ (31)

where ΔT is the updating time of the PF model.

The post NOx sensor and ammonia sensor reading are selected for the measurement equation which can be shown as follows:

$$z(k)=h[\hat{x}(k)]=\left[\begin{array}{c} C_{\mathrm{NO}}(k)\\ C_{{\mathrm{NH}}_{3}}(k) \end{array}\right]=\left[\begin{array}{c} {\hat{C}}_{\mathrm{NO}}(k‑1)+\frac{1}{2}∆T[{\dot{\hat{C}}}_{\mathrm{NO}}(k)+{\dot{\hat{C}}}_{\mathrm{NO}}(k‑1)\\ {\hat{C}}_{{\mathrm{NH}}_{3}}(k‑1)+\frac{1}{2}∆T[{\dot{\hat{C}}}_{{\mathrm{NH}}_{3}}(k)+{\dot{\hat{C}}}_{{\mathrm{NH}}_{3}}(k‑1) \end{array}\right]$$ (32)

where

$${\dot{\hat{C}}}_{\mathrm{NO}}(k)=-C_{\mathrm{NO}}\left[\Theta r_{\mathrm{re}}{\hat{\theta }}_{{\mathrm{NH}}_{3}}(k|k‑1)+\frac{F}{V}\right]+r_{\mathrm{ox}}\Theta {\hat{\theta }}_{{\mathrm{NH}}_{3}}(k|k‑1)+\frac{F}{V}C_{\mathrm{NO},\mathrm{in}}$$ (33)

$${\dot{\hat{C}}}_{{\mathrm{NH}}_{3}}(k)=-C_{{\mathrm{NH}}_{3}}\left[\Theta r_{\mathrm{ad}}(1‑{\hat{\theta }}_{{\mathrm{NH}}_{3}}(k|k‑1))+\frac{F}{V}\right]+r_{\mathrm{de}}\Theta {\hat{\theta }}_{{\mathrm{NH}}_{3}}(k|k‑1)+\frac{F}{V}{\hat{C}}_{{\mathrm{NH}}_{3},\mathrm{in}}(k|k‑1)$$ (34)

where C_NO,in_ is the NOx input concentration value measured by physical NOx sensor placed before the SCR Can.

***Remarks*:** In the observer designed, two NOx sensors placed at the entrance of the SCR Can and the tailpipe respectively are needed and one ammonia sensor located at the tailpipe is also necessary. It is worth noting that most onboard NOx sensors have significant ammonia cross sensitivity which would make the observation unsatisfactory if the inaccurate NOx sensor reading is used. However, by using the measurement value of ammonia sensor and temperature sensor in the tailpipe, the NOx sensor reading can be corrected to eliminate the effect of gaseous ammonia species. In recent years, a lot of approaches have been proposed to obtain the corrected NOx sensor[36, 37]. In this paper, a temperature-dependent cross-sensitivity factor is used to correct the NOx sensor values.

## Simulation results and comparisons

The parameters of the SCR system model established above are derived from the previous research results in [38]. With these identified parameters, the PF observer is designed and the simulations and comparisons are carried out based on an experimentally-validated full vehicle simulator cX-Emission exploited by the Center for Automotive Research at the Ohio State University[12]. The full vehicle simulator cX-Emission is developed by using the Model-Based Design in MATLAB/Simulink and the architecture of the developed full vehicle simulator is selected that utilizes a 1.9 L Diesel-engine belted to a 10.6kW starter/alternator; see Ref [39].

For the simulation, as we can see in the SCR system model in Eq (17), the exhaust gas flow rate, exhaust temperature and engine-out NOx concentration are all required. For these information, the city driving cycle FTP-75 is used which is a test cycle released by the US Environmental Protection Agency(EPA) to measure the tailpipe emissions and fuel economy. According to the cX-Emission simulator and the FTP-75 test cycle, the exhaust flow rate, exhaust temperature, engine-out NOx concentration and input ammonia concentration of SCR system during the test are presented as follows.

**Fig 3:**
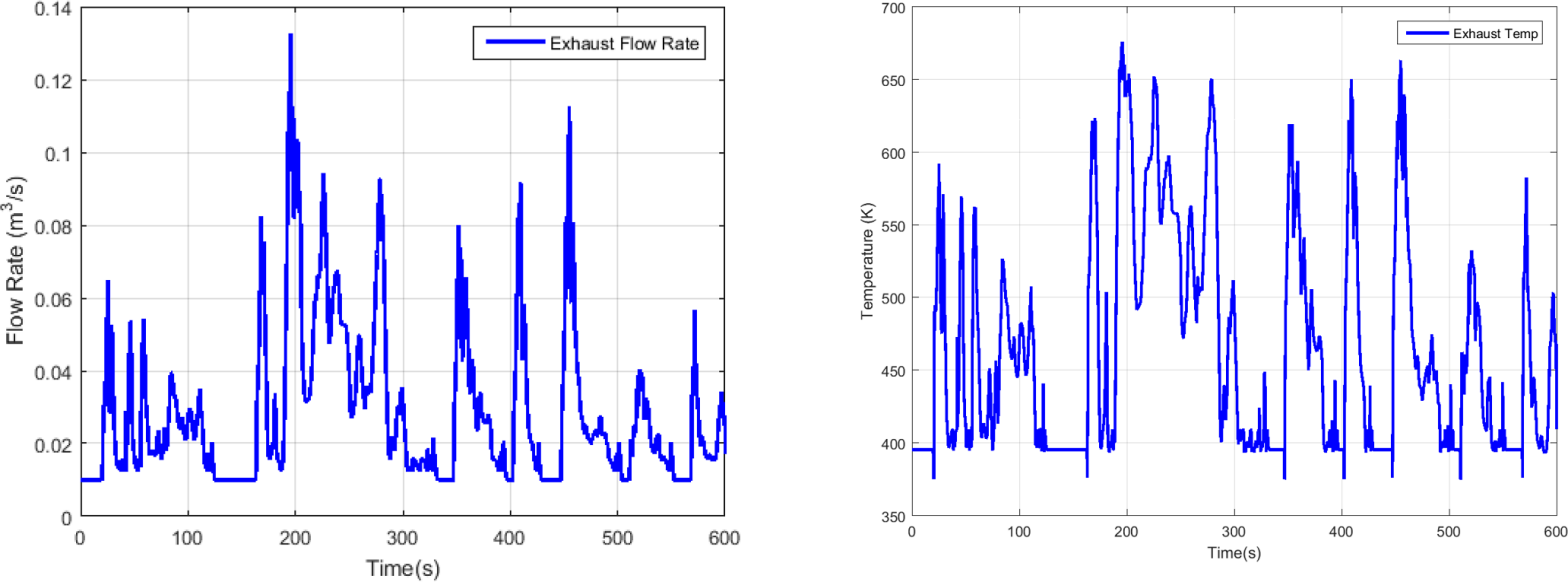
Exhaust flow rate and exhaust temperature during the FTP-75 test cycle simulation.

**Fig 4:**
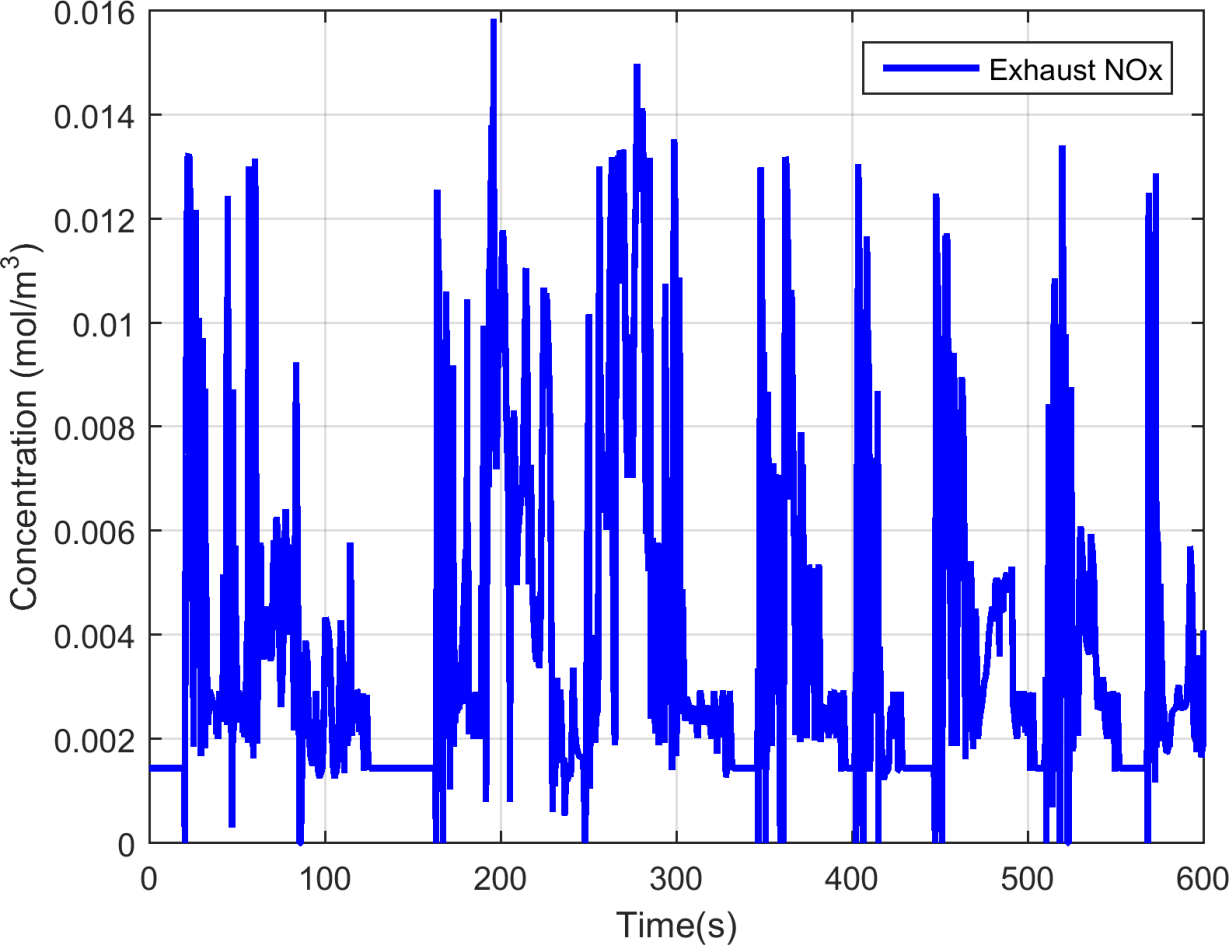
The engine-out NOx concentration during the FTP-75 test cycle simulation.

**Fig 5:**
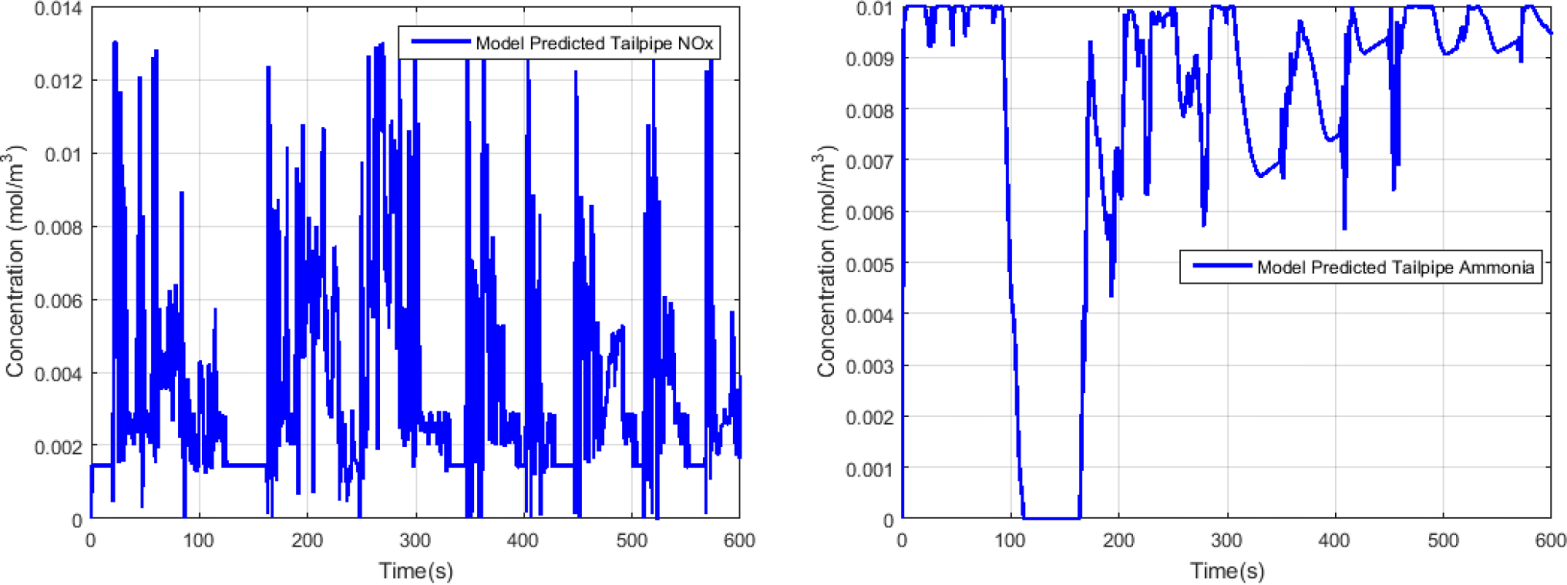
The model predicted tailpipe NOx and ammonia concentration.

Fig 3 shows the exhaust gas flow rate and temperature during the FTP-75 test cycle. We can see that the flow rate and temperature vary a lot which is representative for the real-world applications. Fig 4 depicts the engine-out NOx concentration which is the sum of the NO and NO_2_ concentrations. The model predicted tailpipe NOx and ammonia concentration are presented in Fig 5. Compared with the engine-out NOx concentration, the tailpipe NOx concentration does not decrease significantly, that is because the temperature is low, the reduction rate is slow. The exhaust temperature is low in the period of 110s to 160s, resulting in no urea injection in order to avoid urea crystallization, and this also leads to a significant reduction in the concentration of tailpipe ammonia.

**Fig 6:**
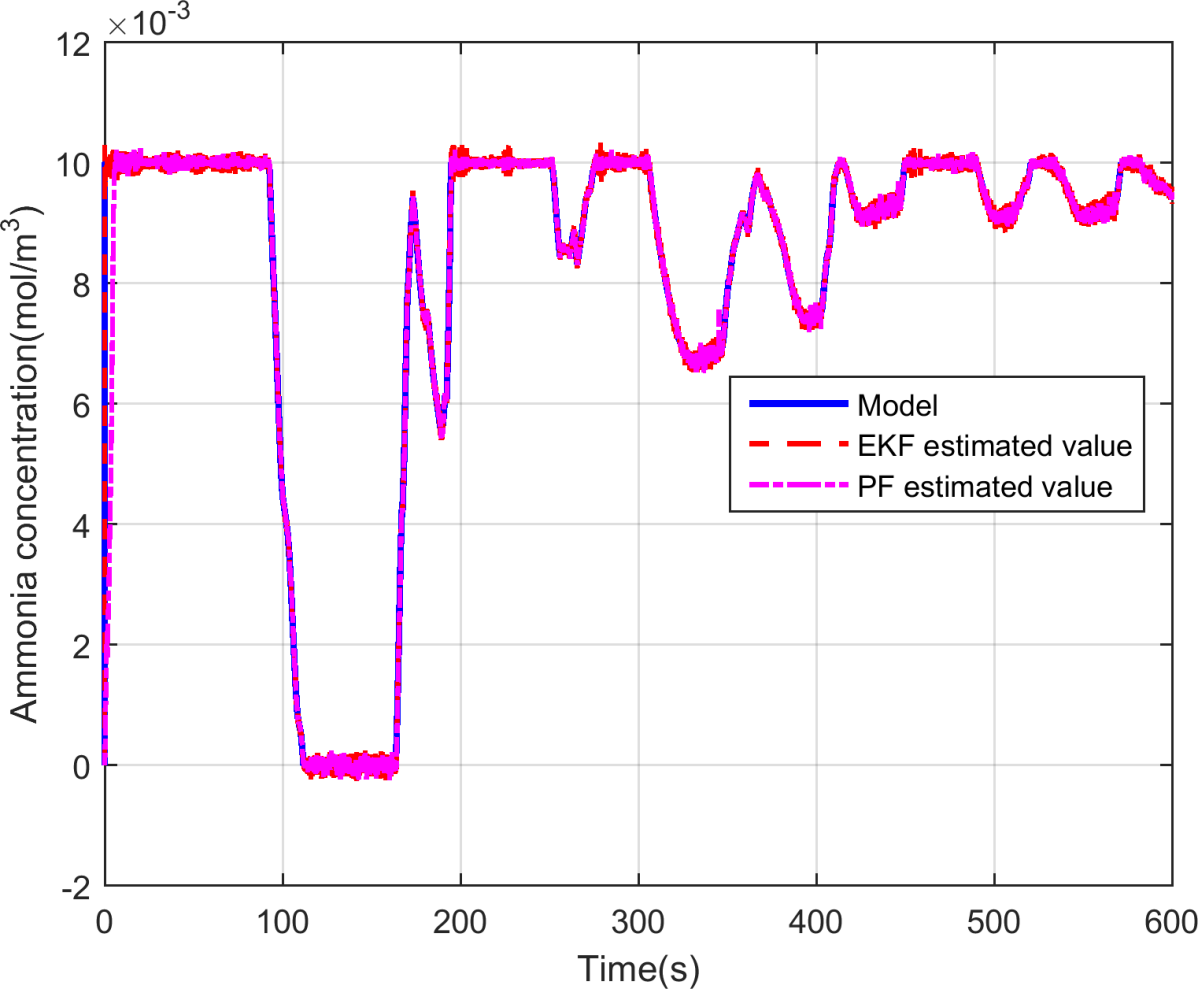
Comparisons of model predicted ammonia concentration and PF estimated ammonia concentration.

**Fig 7:**
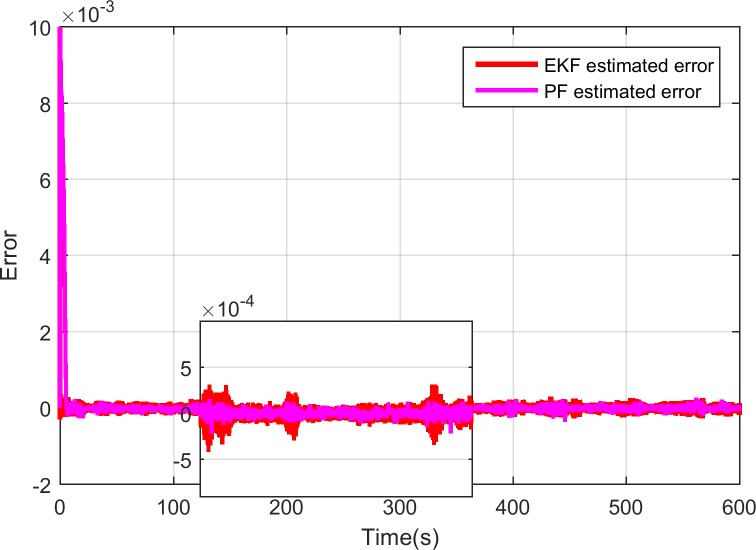
Errors of model predicted ammonia concentration and PF estimated ammonia concentration.

As above-presented, the designed input ammonia concentration and ammonia coverage ratio observer based on PF is simulated based on the selected FTP-75 cycle. Fig 6 depicts the input ammonia concentration comparison during the test. The solid-blue line is the input ammonia concentration which is obtained based on the PID control law in [40]. The red and pink curve shows the estimated ammonia concentration by the designed EKF and PF observer respectively. As can be seen from Fig 6, the observations quickly respond to the calculated values of the SCR model and has a good traceability where the calculated values change. Fig 7 shows the errors between the observer estimation and the SCR model predicted value. The error is defined as the model predicted value minus the observer estimated value. However, compared with EKF-based observer, as shown in Fig 7, the PF observer get the smaller errors which are quite small being of the order of 10^−4^. Therefore, as expected, we can claim that the proposed estimation method based on PF algorithm is more accurate for estimating the input ammonia concentration and can meet the actual needs.

**Fig 8:**
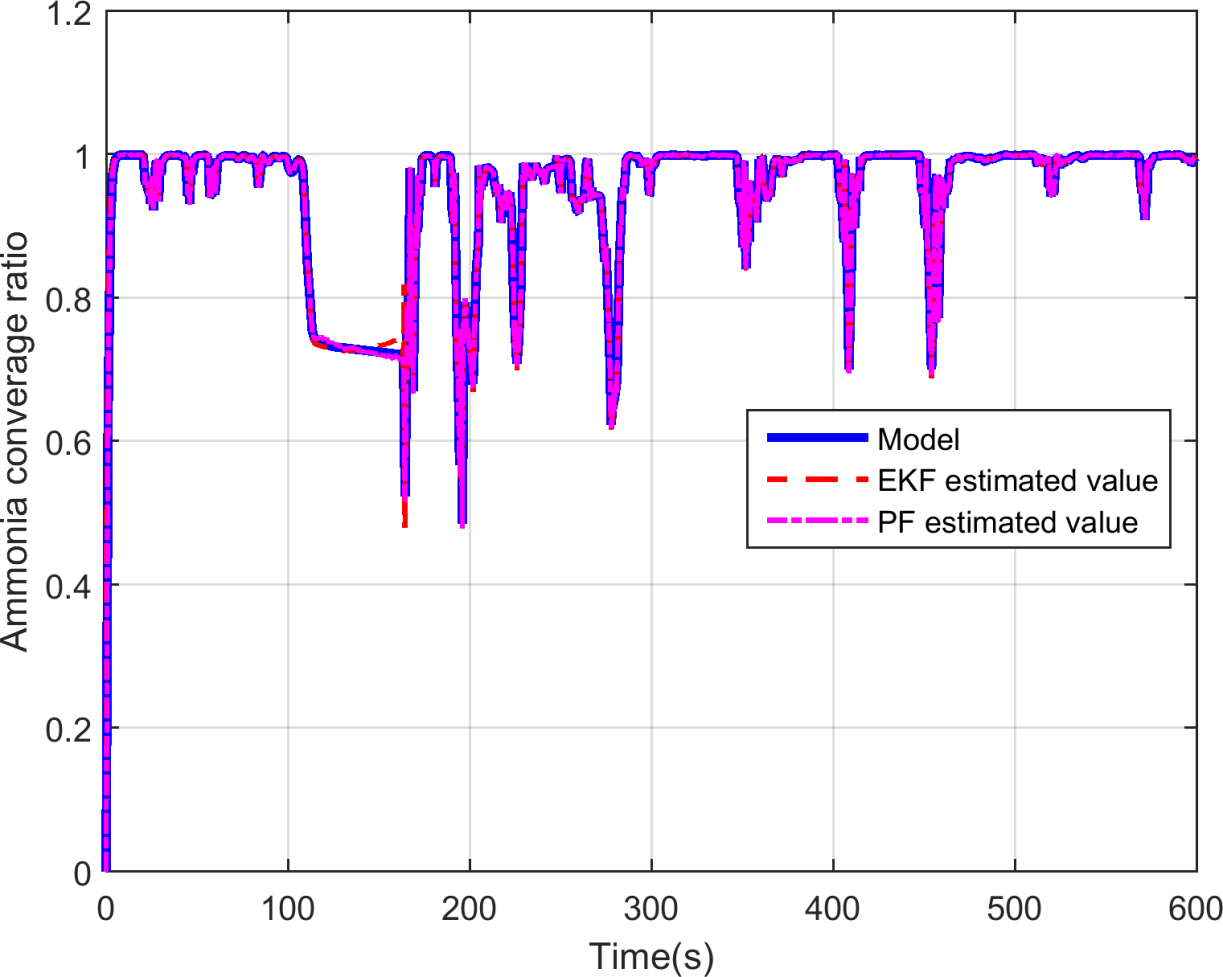
Comparison of model predicted ammonia coverage ratio and PF estimated ammonia coverage ratio.

**Fig 9:**
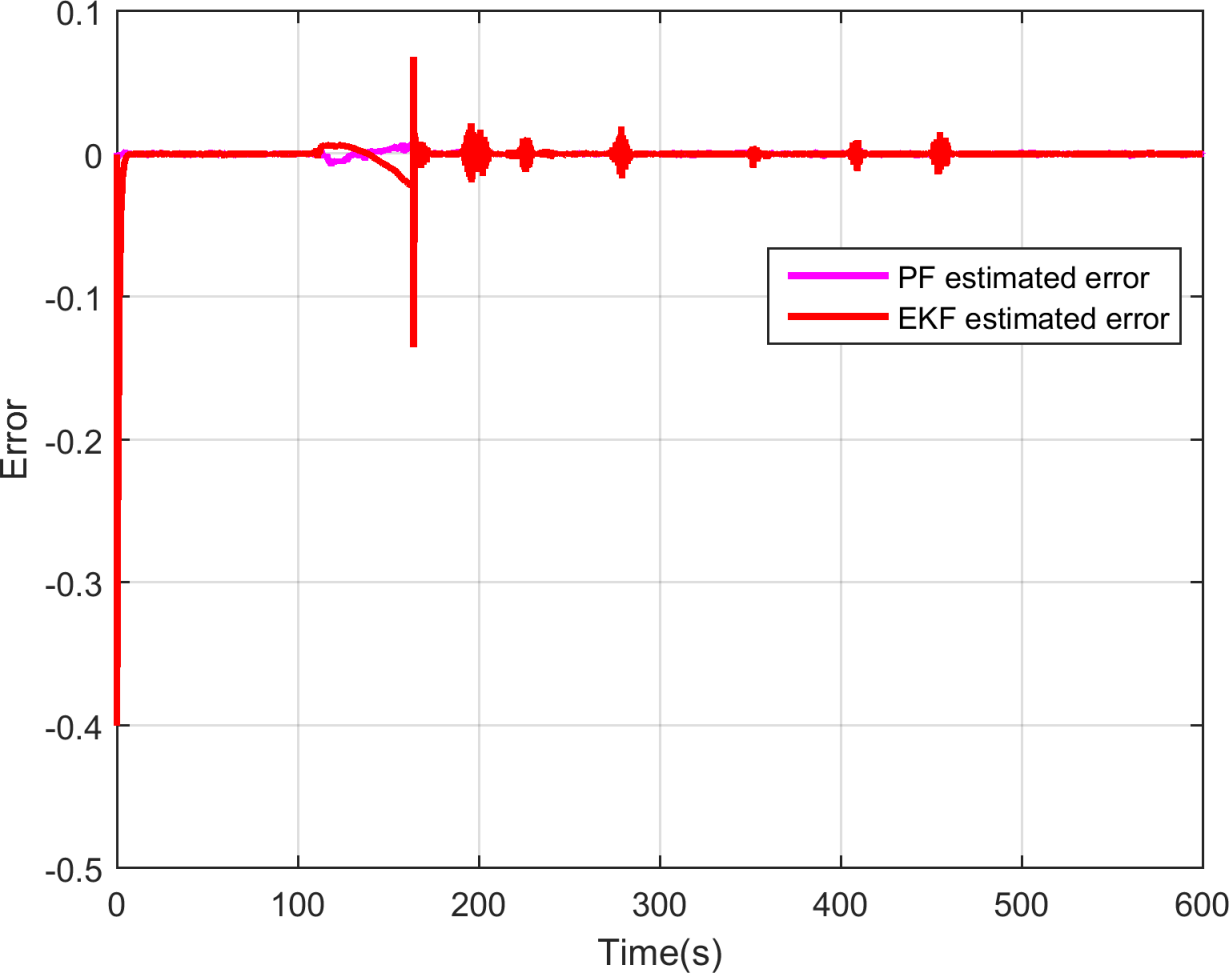
Errors of model predicted ammonia coverage ratio and PF estimated ammonia coverage ratio.

As is mentioned above, the ammonia coverage ratio cannot be directly measured by a physical sensor. Thus, we can only compare the ammonia coverage ratio values between the SCR model and the designed observers. And Fig 8 presents the comparison of the SCR model calculated ammonia coverage ratio and the estimated one by the developed EKF and PF observers where we can see that the estimated ammonia coverage ratio by PF observer can better approach to the one of the SCR model than the one by EKF, especially at around 180s. Fig 9 is the error, and the error is defined as the model calculated value minus the observer estimated value. As is shown in the figure, the errors induced by EKF observer are severe at some time. On the contrary, though the ammonia coverage ratio calculated by the SCR model changes violently, the PF observer can catch up with it quickly and the errors depicted in Fig 9 are quite small which shows the performance of the designed observer based on PF algorithm works better for the SCR system.

## Conclusions

The PF observer for input ammonia concentration and ammonia coverage ratio estimations is studied in this paper. Though the input ammonia concentration value can be measured by ammonia sensors, this method will increase the cost and diagnosis challenge of the SCR device. Inspired by the problem, the PF observer is designed to replace the physical sensors. According to the comparison results, the performance of the designed observer based on PF is excellent during the FTP-75 test cycle. Therefore, with the assistance of the developed observer, the ammonia sensor placed at the entrance of the SCR tank can be cancelled. What’s more, the ammonia coverage ratio information is critical in the construction of the feedback control strategy and cannot be measured directly by physical sensor. And the simulation results and comparisons show that the designed PF observer has a pretty good ability to estimate the ammonia coverage ratio value.

In the future research, more experiments for Diesel-engine and SCR system will be done gradually, and other kinds of estimation methods will be considered. Besides, the closed-loop control strategy based on ammonia coverage ratio and other crucial sates will be designed.

## Supporting information

S1 File The data file. (MAT)
